# *NBN* rs1805794 Polymorphism Increases the Predictive Performance of Machine Learning Models for Multiple Chronic Toxicities in Head and Neck Cancer Survivors Treated with Definitive Radiotherapy ± Chemotherapy

**DOI:** 10.3390/jcm15166264

**Published:** 2026-08-13

**Authors:** Sevda Yener, Seda Ekizoglu, Meltem Dağdelen, Gökçen Civan, Fırat Tevetoğlu, Zeliha Kübra Çakan, Ayşe Çırakoğlu, Ömer Erol Uzel

**Affiliations:** 1 Department of Radiation Oncology, Bağcılar Training and Research Hospital, 34164 Istanbul, Turkey; 2 Department of Medical Biology, Cerrahpaşa Medical Faculty, Istanbul University-Cerrahpaşa, 34098 Istanbul, Turkey; seda.ekizoglueratak@iuc.edu.tr (S.E.); ayse.cirakoglu@iuc.edu.tr (A.Ç.); 3 Department of Radiation Oncology, Cerrahpaşa Medical Faculty, Istanbul University-Cerrahpaşa, 34098 Istanbul, Turkey; meltem.dagdelen@iuc.edu.tr (M.D.); ouzel@iuc.edu.tr (Ö.E.U.); 4 Department of Radiology, Beykoz State Hospital, 34800 Istanbul, Turkey; gokcen132@gmail.com; 5 Department of Otorhinolaryngology, Cerrahpaşa Medical Faculty, Istanbul University-Cerrahpaşa, 34098 Istanbul, Turkey; firat.tevetoglu@iuc.edu.tr; 6 Department of Medical Oncology, Cerrahpaşa Medical Faculty, Istanbul University-Cerrahpaşa, 34098 Istanbul, Turkey; zeliha.cakan@iuc.edu.tr

**Keywords:** radiotherapy, multiple chronic toxicities, NBN, rs1805794, machine learning

## Abstract

**Objective:** While advancements in radiotherapy and systemic agents have significantly improved survival rates in head and neck squamous cell carcinoma (HNSCC), managing long-term, treatment-induced toxicities remains a critical clinical challenge. This study aimed to develop a personalized, supervised machine learning-driven predictive model for multiple chronic toxicities by integrating clinical, dosimetric, and genetic data specifically evaluating the impact of the *NBN* gene rs1805794 (c.553G>C) polymorphism. **Methods:** This study enrolled 125 patients with HNSCC who received curative-intent radiotherapy and remained disease-free during follow-up with a median of 98 months. Comprehensive clinical and dosimetric data were collected, and chronic toxicities were recorded. Peripheral blood samples were analyzed for the *NBN* rs1805794 polymorphism using allele-specific PCR (AS-PCR). Following feature selection, four supervised machine learning classifiers were trained and evaluated to identify the optimal model for predicting multiple chronic toxicities. **Results:** The XGBoost algorithm emerged as the highest performing model. Baseline clinico-dosimetric predictors of multiple chronic toxicities included PTV70 volume, the addition of concurrent chemotherapy, advanced T and N stages, and continued smoking. Integrating the *NBN* rs1805794 genotype into the XGBoost architecture enhances its predictive capability. The final model accurately identified patients at high risk for multiple chronic toxicities, achieving an area under the curve (AUC) of 0.78, an accuracy of 0.77, a sensitivity of 0.74, and a specificity of 0.79. **Conclusions:** Integrating clinical, dosimetric, and genetic data within a machine learning framework effectively predicts multiple chronic toxicities in HNSCC. This approach enabled early risk stratification, providing the potential for personalized therapy.

## 1. Introduction

Approximately 660,000 new head and neck cancers (HNCs) are diagnosed worldwide each year, and these malignancies are responsible for approximately 325,000 deaths annually [1,2]. In recent years, survival rates have improved significantly due to advances in systemic treatments and radiotherapy (RT) techniques such as intensity-modulated radiotherapy (IMRT), as well as the increasing incidence of human papillomavirus (HPV)-associated oropharyngeal cancer, which has a more favorable prognosis [3]. Although IMRT reduces treatment-related toxicity, many patients still experience side effects [4,5]. While agents such as bevacizumab, pentoxifylline, vitamin E, or hyperbaric oxygen have demonstrated some efficacy in managing acute side effects, their effectiveness in addressing chronic toxicities remains limited [6,7]. Once chronic toxicity develops, it is generally irreversible and can have a negative physical, social and psychological impact on patients, creating a significant burden on the healthcare system [8]. Therefore, predicting toxicity and taking preventive measures are crucial in radiotherapy planning.

The risk of radiation induced toxicity depends primarily on the radiation dose received by surrounding normal tissues. However, studies in the literature have shown that chemical use, chronic diseases such as diabetes and collagen tissue diseases, and age are important in the development of toxicity [9]. The level of radiation sensitivity differs among patients due to the complex pathophysiology of toxicity development [10]. Predicting radiation toxicity based on patient-specific factors allows more personalized cancer treatment strategies to be developed. Similar oncological outcomes can be achieved by safely increasing radiotherapy doses in patients at low risk of toxicity. Conversely, patients identified as high risk for severe toxicities may benefit from alternative therapies, such as surgery or proton therapy, or from radiotherapy planning that strictly prioritizes sparing normal tissue.

Several clinical, genetic, and dose volume histogram (DVH)-derived factors have been studied to predict radiation-induced toxicity. Higher radiation doses, larger irradiated volumes, and the addition of chemotherapy to radiotherapy are known to increase treatment-related toxicity [10,11]. In addition, genetic susceptibility for development of toxicities is important. A genome-wide association study (GWAS) investigating the long-term side effects experienced by 352 patients with oropharyngeal cancer revealed that the rs11714564 variant of the *RTP1* (receptor transporter protein 1) gene increases the risk of severe xerostomia following radiotherapy [12].

The *NBN* gene (also known as cell cycle regulatory protein p95; Nijmegen breakage syndrome protein 1), located on human chromosome 8q21.3, encodes the 754 amino acid Nibrin protein (UniProt ID: O60934). Nibrin is a component of the MRE11-RAD50-NBN (MRN) complex, which plays a central role in double-strand DNA break repair, DNA recombination, telomere maintenance, and meiosis. The NBN protein carries a nuclear localization signal (NLS) and facilitates the transport of the MRN complex to the nucleus when DNA is damaged in both strands. It also regulates the catalytic activities of MRE11 and RAD50 in the presence of ATP. On the other hand, NBN is involved in calling ATM, a serine/threonine kinase, to the damaged area. ATM is activated by autophosphorylation and initiates the DNA damage response by phosphorylating proteins involved in DNA repair, such as p53, H2AX, and CHK2. ATM also phosphorylates proteins in the MRN complex, thereby increasing MRN complex stability at DNA double-strand breaks, promoting checkpoint activation, facilitating homologous recombination repair, and ensuring efficient nuclear localization of the complex [13].

Alterations in the *NBN* gene directly affect the cell’s DNA repair capacity by altering protein structure and function. The most common mutation in the *NBN* gene is c.657_661del5, resulting in the inability to repair DNA damage and Nijmegen breakage syndrome (NBS) [14,15]. In contrast, common polymorphisms in the *NBN* gene, such as E185Q (rs1805794) and I171V (rs61753720), alter protein conformation, reducing the stability of the MRN complex and its ability to repair double-strand breaks in DNA. This structural defect does not cause complete loss of function but leads to cellular radiation sensitivity [16]. Furthermore, these polymorphisms have been associated with susceptibility to various malignancies, including breast, leukemia, and nasopharyngeal cancer. While MRE11 and RAD50 have been relatively extensively studied in relation to radiosensitivity and treatment-related toxicity, NBN has received comparatively less attention despite its equally critical role within the same repair complex. Given this relative gap in the literature, we selected the *NBN* rs1805794 polymorphism to investigate its potential contribution to chronic radiotherapy-induced toxicity in HNC patients [17,18,19].

Traditional statistical methods may be inadequate for high-dimensional datasets due to the complexity of the modeling assumptions and dimensionality issues [20]. Machine learning provides more flexible and powerful modeling capabilities, especially in high-dimensional datasets [21]. Consequently, there has been a marked increase in the application of artificial intelligence models in the field of cancer research [22,23,24,25,26].

In recent years, several artificial intelligence models have been developed to predict radiotherapy-induced toxicities in HNC patients, most of which rely predominantly on clinical and dosimetric parameters. In the study by Dean et al., a machine learning-based predictive model was developed to identify patients at risk of severe dysphagia. The model showed good predictive performance, with an AUC of 0.82 for severe dysphagia prediction [27]. Furthermore, it has been shown that deep learning models are accurate at predicting xerostomia in an RTOG trial [28]. Beyond clinical and dosimetric variables, the integration of genetic data into toxicity prediction models has been explored mainly outside the HNC setting. In prostate cancer, the combination of genetic and dosimetric variables provided a significant improvement in NTCP prediction using both analytical and data-driven approaches [29]. In the WECARE study, the risk of radiation therapy-induced contralateral secondary breast cancer was investigated using genome-wide association study (GWAS) data. A machine learning model incorporating single-nucleotide polymorphisms demonstrated modest predictive performance, achieving an AUC of 0.62 for predicting radiation therapy-induced contralateral breast cancer [30]. However, these studies either relied solely on clinical and dosimetric predictors or, when genetic data were included, were conducted outside the HNC setting. Combining genetic, clinical, and dosimetric variables within a machine learning framework for chronic toxicity prediction in HNC survivors remains largely unexplored, and the present study aims to contribute to this area.

The primary objective of this study was to develop a predictive supervised machine learning model integrating clinical, dosimetric, and genetic data. Initially, a baseline algorithm was constructed utilizing clinical and dosimetric parameters alone. Subsequently, we evaluated the impact of incorporating the *NBN* rs1805794 polymorphism to determine its effect on the model’s overall predictive performance.

## 2. Materials and Methods

### 2.1. Study Population

This study enrolled patients diagnosed with head and neck squamous cell carcinoma (HNSCC) who received curative-intent radiotherapy, with or without concurrent chemotherapy, at the Department of Radiation Oncology, Cerrahpaşa Faculty of Medicine, Istanbul University-Cerrahpaşa as part of their standard clinical care and were continuing routine long term follow-up visits at the time of study enrollment. A minimum follow-up period of one year was required for inclusion.

Target volumes were delineated according to MRI, clinical examination, and PET-CT findings. The gross tumor volume (GTV) was contoured based on these findings, and a 5 mm margin was applied and edited according to anatomical boundaries and normal tissue constraints to generate clinical target volumes (CTVs) of 70. A 5 mm margin was subsequently added to CTV70, taking anatomical barriers into account, to define CTV60, representing the intermediate risk volume. A further 5 mm margin was added to CTV60 to generate CTV54, which encompassed the elective nodal volumes. A uniform 3 mm margin was applied to each CTV to generate the corresponding planning target volumes (PTV70, PTV60, and PTV54).

During routine follow-up visits, written informed consent for participation in the present study was obtained from eligible patients. Blood samples for genetic analysis were subsequently collected only from consenting patients, and genetic testing (*NBN* rs1805794 genotyping) was performed thereafter. Patients with a history of prior head and neck surgery or those who developed tumor recurrence during the follow-up period were excluded. The study protocol was reviewed and approved by the Clinical Research Ethics Committee of the Cerrahpaşa Faculty of Medicine, Istanbul University-Cerrahpaşa (Approval No: 652592; Date: 27 March 2023).

### 2.2. Toxicity Evaluation

The physical and endoscopic follow-up examinations were performed every three months during the first two years after treatment, every six months between years 2 and 5, and annually after five years. Contrast-enhanced neck MRI and, in cases of doubt, positron emission tomography (PET)-CT were used for radiological assessment. Patients were questioned about chronic side effects at each visit.

Hearing loss, dysphagia, dysgeusia, xerostomia, neck fibrosis, trismus, osteoradionecrosis, cranial nerve dysfunction, hypothyroidism, dental caries, chronic otitis media, hypopituitarism, aspiration, visual disturbance, dysphonia and carotid artery stenosis were graded according to CTCAE v5. Patients with grade ≥2 hearing loss, dry mouth, dysphagia, neck fibrosis, trismus, dysgeusia, osteoradionecrosis, cranial nerve dysfunction, hypothyroidism, dental caries, chronic otitis media, hypopituitarism, visual impairment, dysphonia, and carotid artery stenosis were considered symptomatic.

In this study, the primary predictive endpoint was defined as the development of multiple chronic toxicities. This was characterized by the concurrent presence of two or more symptomatic late side effects of Grade ≥2 (according to CTCAE criteria), rather than isolating a single Grade ≥3 event, to better reflect the cumulative late morbidity experienced by the patients.

Patients with two or more symptomatic side effects were defined as having severe toxicity and were used as the binary endpoint of this study.

### 2.3. NBN rs1805794 Polymorphism Analysis

During routine outpatient clinic visits, 5 cc of peripheral venous blood was collected from each patient into ethylenediaminetetraacetic acid (EDTA) tubes. Genomic DNA was extracted via an ExtractMe Genomic DNA Kit (Cat.No.EM13; BLIRT S.A., Gdansk, Poland) and the concentration and purity of the isolated DNA were measured via a NanoPhotometer N60 (Implen GmbH, Munich, Germany).

The MANE Select transcript of the *NBN* gene (NM_002485.5; ENST00000265433.8) was used for variant annotation. The rs1805794 variant was analyzed via allele-specific polymerase chain reaction (AS-PCR) on the CFX96 Touch Real-Time PCR Detection System (Bio-Rad Laboratories, Hercules, CA, USA), and nucleotide changes were identified by melting curve analysis.

To detect the G>C substitution in rs1805794, two parallel PCRs were performed for each genomic DNA sample via allele-specific forward primers (G-allele-specific and C-allele-specific). A common reverse primer was used for both reactions. The allele-specific forward primer sequences were 5′-AGGCTGCTTCTTGGAATC-3′ (Gallele) and 5′-AGGCTGCTTCTTGGAATG-3′ (Callele), whereas the common reverse primer sequence was 5′-ATTGTAGACAATATGTGCACTCA-3′. PCR amplification was performed in a reaction volume of 20 µL containing 1× EvaGreen-containing master mix, 0.5 µM of each primer, and 1.5–15 ng of template DNA. The reaction conditions consisted of initial denaturation at 95 °C for 15 min, followed by 44 cycles of denaturation at 95 °C for 15 s and annealing/extension at 57 °C for 40 s, with subsequent melting curve analysis.

### 2.4. Data Analysis Workflow and Feature Selection

A comprehensive panel of 28 features was evaluated for model development, comprising 13 clinical, 14 dosimetric, and one genetic variable.

Clinical Features: Age, sex, smoking history, current smoking status, diabetes mellitus, tumor anatomical subsite, T-stage, N-stage, treatment regimen (induction, concurrent, or induction plus concurrent), planning target volumes (PTV70, PTV60, PTV54), and radiotherapy technique (simultaneous integrated boost [SIB] or sequential).

Dosimetric Features: Mean radiation doses to the oral cavity, right and left submandibular glands, right and left parotid glands, right and left temporomandibular joints, pharyngeal constrictor muscles (mean and maximum), esophageal maximum dose, larynx mean dose, thyroid mean dose, and right and left cochlear mean doses.

Genetic Feature: The *NBN* rs1805794 polymorphism.

#### 2.4.1. Cross-Validation Strategy

**Figure 1:**
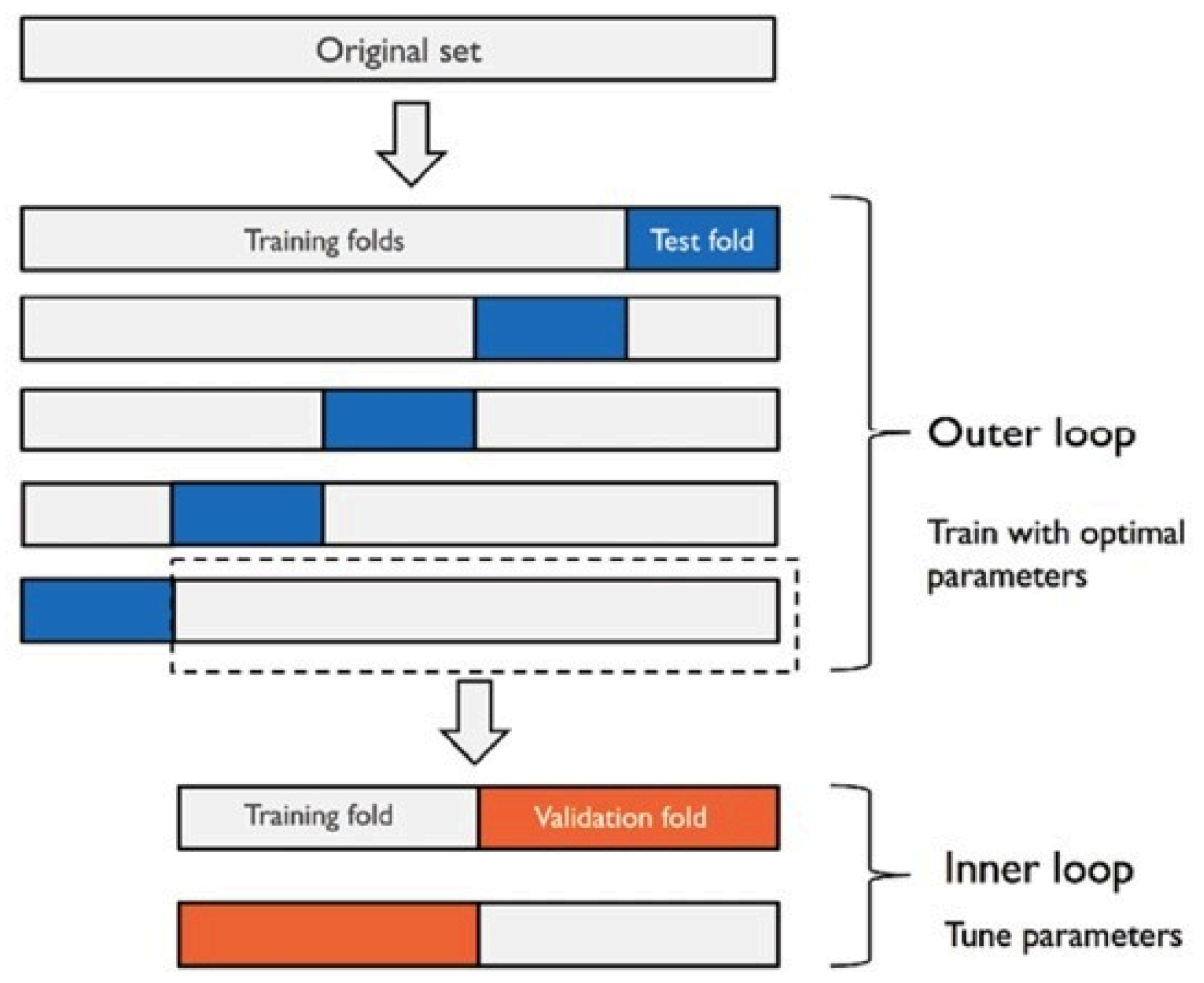
Workflow of the k-fold nested cross-validation analysis.

Given the single-center nature of the cohort and the relatively limited sample size (n = 125), a rigorous nested cross-validation (CV) strategy was implemented to prevent data leakage, mitigate overfitting, and ensure unbiased model evaluation. The architecture consisted of an outer loop for final performance evaluation and an inner loop for feature selection and hyperparameter tuning. Specifically, a 5-fold repeated stratified k-fold CV was utilized in the outer loop. Within each outer fold, an inner 5-fold nested k-fold CV was executed to partition the training data further into smaller training and validation subsets. This inner loop identified the optimal hyperparameter configurations and best-performing feature subsets. The final predictive performance of the models was exclusively derived from the unseen test sets generated by the outer loop iterations (25 patients per test set). This entire process was repeated five times to yield mean performance metrics and standard deviations. The complete analytical pipeline is illustrated in Figure 1.

#### 2.4.2. Hyperparameter Optimization and Missing Data Handling

Hyperparameter optimization was conducted using an exhaustive grid search within the five-fold inner loop of the nested cross-validation procedure, with mean ROC-AUC used as the optimization criterion. The parameters evaluated were the regularization strength and penalty type for logistic regression; the regularization parameter for the linear support vector machine; the number of trees, maximum tree depth, minimum samples required for splitting, and minimum leaf size for random forest; and the number of boosting trees, maximum tree depth, learning rate, subsampling ratio, and column-sampling ratio for XGBoost. The optimal configuration identified in the inner loop was refitted on the complete outer training fold and evaluated exclusively on the held-out outer test fold. Missing data were imputed using five-nearest-neighbor imputation. Numerical variables were standardized, and categorical variables were one-hot encoded. All preprocessing procedures, including imputation, scaling, encoding, and feature selection, were fitted exclusively using the training data within each cross-validation fold and subsequently applied to the corresponding validation or test data to avoid information leakage.

### 2.5. Model Development and Interpretability

Feature selection was performed separately within each inner cross-validation training fold. First, Spearman correlation analysis was used to identify highly correlated predictors. For strongly correlated feature pairs, the variable with the lower predictive relevance was excluded. Mutual-information analysis was then applied to the remaining variables to quantify their predictive associations with the outcome. Both correlation filtering and mutual-information scores were recalculated within each training fold. Therefore, no information from the inner validation folds or outer test folds was used during feature selection, thereby minimizing the risk of data leakage.

Four classifiers were evaluated: logistic regression, a support vector machine with a linear kernel, random forest, and XGBoost. Hyperparameter optimization was performed using an exhaustive grid-search strategy within the five-fold inner cross-validation loop. Mean ROC-AUC across the inner validation folds was used as the optimization criterion. The following hyperparameters were evaluated:

- For logistic regression, the regularization strength (C: 0.01, 0.1, 1, 10, and 100) and penalty type (L1 and L2) were optimized.
- For the linear support vector machine, the regularization parameter (C: 0.01, 0.1, 1, 10, and 100) was optimized.
- For random forest, the number of trees (n_estimators: 100, 300, and 500), maximum tree depth (max_depth: None, 5, 10, and 20), minimum number of samples required to split an internal node (min_samples_split: 2, 5, and 10), and minimum number of samples required at a terminal leaf (min_samples_leaf: 1, 2, and 4) were optimized.
- For XGBoost, the number of boosting trees (n_estimators: 100, 300, and 500), maximum tree depth (max_depth: 3, 5, and 7), learning rate (learning_rate: 0.01, 0.05, and 0.1), subsampling ratio (subsample: 0.7, 0.9, and 1.0), and column-sampling ratio (colsample_bytree: 0.7, 0.9, and 1.0) were optimized.

For each outer iteration, the combination of preprocessing parameters, selected features, and classifier hyperparameters that yielded the highest mean inner-fold ROC-AUC was selected. The complete optimized pipeline was subsequently refitted using the entire outer training set and evaluated once on the corresponding held-out outer test set. Out-of-fold predictions from the outer test folds were combined to calculate the final discrimination and calibration measures.

To ensure transparency and interpretability of the highest-performing XGBoost model, partial dependence plots (PDPs) were generated to visualize the marginal effect of individual features on the model’s predictive output. Finally, to evaluate the incremental value of the *NBN* polymorphism, we developed paired models with and without RS using identical cross-validation splits. The AUC difference and its 95% confidence interval were estimated from paired out-of-fold predictions using 2000 stratified bootstrap resamples, with a two-sided bootstrap *p*-value testing whether the AUC difference differed from zero.

## 3. Results

### 3.1. Patient Characteristics and Clinical Outcomes

A total of 125 patients were included in the final analysis. The mean age of the study cohort was 50 years (range: 18–78 years), with a male predominance (n = 92, 73.6%). The baseline demographic and clinical characteristics of the patients are summarized in Table 1, and the detailed dosimetric parameters are provided in Table 2. Over a median follow-up period of 98 months (range: 13–295 months), 61 patients (48.8%) developed severe treatment-related toxicity. A detailed analysis of these chronic adverse effects is presented in Table 3.

### 3.2. Genomic Analysis

Genotype and allele frequencies for the *NBN* rs1805794 polymorphism are detailed in Table 4. The frequency of the G allele was 0.632, while the C allele frequency was 0.368. The observed genotype distributions were consistent with Hardy-Weinberg equilibrium (χ^2^ = 0.17; *p* = 0.680).

### 3.3. Feature Selection and Dimensionality Reduction

During the initial variable selection phase, correlation analysis identified strong associations between normal tissue doses and multiple planning target volumes (PTV60, PTV54, and PTV70), indicating a high risk of multicollinearity. Consequently, only PTV70 was retained for further analysis.

Subsequent mutual-information (MI) analysis was conducted to quantify the predictive value of the remaining clinical and dosimetric variables. PTV70 volume (MI: 0.11) and treatment schedule (MI: 0.77) demonstrated the highest information gain. Conversely, sex, primary tumor location, radiotherapy technique, diabetes mellitus, and smoking history exhibited weak associations with severe toxicity (MI < 0.02) and were therefore excluded from the final predictive modeling.

### 3.4. Machine Learning Model Performance

Four distinct machine learning classifiers, logistic regression, linear support vector machine (SVM), random forest, and XGBoost, were trained using the high-MI variables. The baseline performance metrics for these clinico-dosimetric models are presented in Table 5.

Following baseline evaluations, the *NBN* rs1805794 genomic data was integrated to assess its additive predictive value (Table 6). Adding *NBN* rs1805794 genomic data improved discrimination across all four classifiers (ΔAUC: logistic regression +0.047, linear SVM +0.057, random forest +0.063, XGBoost +0.076). For XGBoost, the best-performing model, AUC increased from 0.705 (95% CI: 0.615–0.788) to 0.781 (95% CI: 0.699–0.851), a statistically significant improvement of 0.076 (95% CI: 0.021–0.134; *p* = 0.008).

Calibration also improved for the XGBoost model after including RS: the Brier score decreased from 0.218 to 0.191, the calibration intercept improved from −0.06 to −0.02, and the calibration slope increased from 0.81 to 0.94 (closer to the ideal value of 1.0). Calibration curves visually confirmed closer alignment with the ideal line after *NBN* rs1805794 genomic data inclusion.

### 3.5. Partial Dependence and Risk Probability Analysis

**Figure 2:**
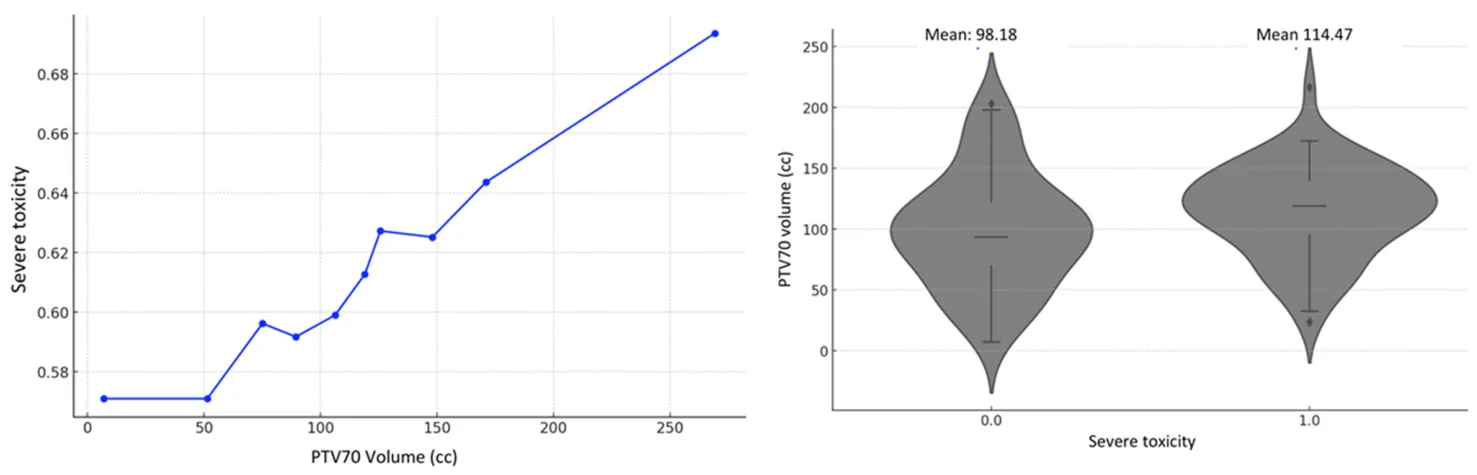
Partial dependence and violin plots illustrating the association between PTV70 volume and severe toxicity.

Analysis of the XGBoost model’s partial dependence plots (PDPs) revealed risk thresholds and decision making patterns:

Dosimetric Factors: The risk of severe toxicity increased concurrently with PTV70 volume. Toxicity was rare in patients with a PTV70 below 98.18 cc, whereas the risk escalated when the volume exceeded 114.47 cc (Figure 2).

Treatment Modality: The baseline risk of severe toxicity in the radiotherapy alone group was 50%; however, this probability increased to 70% with the addition of induction or concurrent chemotherapy.

Tumor Characteristics: Risk probabilities demonstrated a positive correlation with advancing disease stages, increasing from 52% in T1 to 65% in T4 tumors, and from 53% in N0 to 66% in N3 nodal disease.

**Figure 3:**
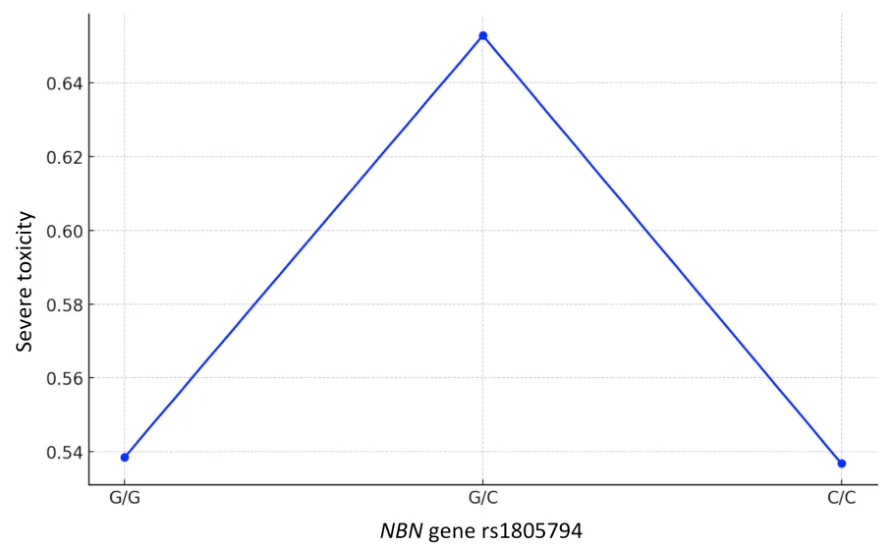
Partial dependence plot for the *NBN* rs1805794 polymorphism.

Genetic Susceptibility: Patients harboring the heterozygous GC genotype exhibited the highest predicted probability of severe toxicity (65.3%), compared to those with the GG (53.8%) and CC (53.7%) genotypes (Figure 3).

Clinical Factors: Continued smoking during treatment increased the absolute likelihood of severe toxicity by 6% (reaching a 60% probability for active smokers). While age and past smoking history influenced toxicity risk, the PDPs indicated that these relationships were nonlinear.

## 4. Discussion

This study developed a machine learning model integrating clinical, dosimetric, and genetic (*NBN* rs1805794 polymorphism) data to predict multiple chronic toxicities in patients with head and neck cancer (HNC). The incorporation of genetic information was associated with improved model performance, suggesting a potential contribution of the *NBN* rs1805794 polymorphism to toxicity risk stratification. The primary predictors of severe toxicity emerged as a PTV70 volume exceeding 114.47 cc, the addition of chemotherapy to radiotherapy, advanced T and N stages, continued smoking, and the presence of the GC genotype.

Radiotherapy-induced toxicity is fundamentally driven by the radiation dose delivered to surrounding normal tissues, which inherently increases with larger tumor volumes [31]. Consistent with this, our analysis identified advanced T and N stages as risk factors, with PTV70 emerging as the most influential predictor in our model. Specifically, in our exploratory analysis, the incidence of multiple chronic toxicities appeared low in patients with a PTV70 below approximately 98 cc, while it increased in patients with a PTV70 above approximately 114 cc. These values should be interpreted as data-driven observations from our cohort rather than validated clinical cut-offs, and require confirmation in independent, prospective studies before informing treatment planning. Nonetheless, these findings are consistent with the broader clinical rationale for precise target delineation, routine use of image-guided radiotherapy (IGRT), and implementation of minimized or personalized PTV margins based on organ motion.

Relying exclusively on dose volume metrics is often insufficient for predicting toxicity due to substantial interpatient variability in inherent radiosensitivity. Interestingly, standard clinical variables such as age, sex, and diabetes mellitus (DM) did not significantly correlate with severe toxicity in our cohort. While DM is a documented risk factor for RT-induced chronic toxicity due to underlying microvascular damage [32,33]; the absence of this association in our study, despite a comparable DM prevalence (16%) in the existing literature [34,35], may be explained by the demographic profile of our cohort. Given the relatively young mean age of the patients, it is possible that the cumulative diabetes-induced vascular damage necessary to exacerbate radiotoxicity had not yet clinically manifested.

Evidence from previous studies suggests that continued smoking during and after treatment is associated with decreased CRT efficacy, poorer tumor control, and an increased incidence of RT-induced toxicity [36]. A previous study reported that the rate of continued smoking among patients treated for laryngeal cancer was 35%, which was higher in patients who underwent RT than in those who underwent surgery [37]. In our study, the continued smoking rate was 24.8%, which was associated with an increased risk of severe toxicity.

Concurrent chemoradiotherapy (CRT) remains the standard of care for locally advanced HNC due to its survival benefits; however, the addition of chemotherapy amplifies toxicity risk. Dong et al. previously demonstrated higher pharyngeal and laryngeal toxicities in CRT cohorts compared to those receiving radiotherapy alone [38]. Interestingly, our study found comparable severe toxicity rates between the induction and concurrent CRT arms, despite the higher overall chemotherapy intensity in the induction group. As suggested by Zhao et al. [39] this may be attributed to our institutional practice of adaptively delineating target volumes based on post-induction tumor regression, thereby mitigating chronic toxicities without compromising local control.

Radiation eradicates tumor cells by inducing single- and double-strand DNA breaks, which are subsequently processed by non-homologous end-joining (NHEJ) or homologous recombination repair (HRR) pathways. Genetic variations (polymorphisms or mutations) in these repair pathways can impair DNA repair capacity in normal tissues, critically influencing the onset of acute and late toxicities by becoming more sensitive to radiation. The *NBN* gene encodes a vital component of the MRN complex within the HRR pathway, and may play a role in radiation-induced toxicity by affecting the repair efficiency of the complex. While the *NBN* rs1805794 polymorphism has been associated with radiotoxicity in breast, non-small cell lung, and prostate cancers [40,41,42,43], data regarding HNC remains sparse. Consistent with the limited available literature, our findings suggest that the *NBN* rs1805794 polymorphism may contribute to individual susceptibility to RT-related toxicity in patients with HNC.

Artificial intelligence has been widely used for predicting radiotherapy-related toxicity in multiple cancer types. Duprez et al. developed a multiparametric model integrating clinical, dosimetric, and genetic data to predict dysphagia in HNC patients, and concurrent CT, the D2 dose to the pharyngeal constrictor muscles and the *XRCC1* gene rs3213245 polymorphism were identified as the most significant predictors, yielding an AUC of 0.60 [44]. Similarly, Wojcieszynski et al. used machine learning to predict toxicity in HNC patients treated by CRT by integrating clinical and dosimetric parameters and reported AUC values of 0.65 for 90-day toxicity and 0.63 for 180-day toxicity, with the PTV integral dose identified as the most significant predictor [45]. By integrating the *NBN* rs1805794 variant into our clinico-dosimetric model, predictive performance improved, supporting the potential value of incorporating genetic information into toxicity prediction models. (AUC 0.781). Although external validation is required, this approach may facilitate the identification of patients at increased risk of long-term treatment-related toxicity, potentially enabling earlier implementation of preventive strategies and more individualized treatment planning.

One of the strengths of this study is the median follow-up duration of 98 months. Given that several late radiation-related toxicities may develop gradually over many years, the extended follow-up period enabled a more complete assessment of chronic treatment-related morbidity. This may be particularly relevant for complications such as fibrosis, xerostomia, and osteoradionecrosis, which are not always fully captured in studies with shorter follow-up durations.

In the current literature, machine learning models for radiotoxicity often focus on predicting an isolated, organ-specific endpoint. We deliberately modeled a composite endpoint rather than organ-specific outcomes because, despite arising from mechanistically distinct pathways, these toxicities share common upstream drivers, irradiated normal tissue volume, concurrent chemotherapy, and impaired DNA damage repair capacity, and because HNC survivors in a densely irradiated anatomical region rarely experience a single isolated complication. This choice inevitably limits the model’s granularity: a high-risk prediction indicates an elevated overall morbidity burden but does not specify which individual toxicity is most likely to occur. Future work using multi-label or multitask modeling frameworks could extend this approach to generate toxicity-specific probabilities alongside the composite risk score.

While the findings of this study are encouraging, several limitations should be considered when interpreting the results. First, the retrospective design and the relatively small sample size (n = 125) from a single institution may limit the broad generalizability of our findings. Although we employed a nested cross-validation strategy to mitigate the risk of overfitting and ensure internal validity, the lack of an independent external validation cohort remains a primary constraint. Also it should be noted that the model’s discriminative performance (AUC = 0.781) is moderate, and caution is warranted when interpreting its potential clinical utility. An AUC in this range indicates that while the model captures a meaningful predictive signal, misclassification remains possible at the individual patient level.

Therefore, the current model should be regarded as a hypothesis-generating tool rather than a validated decision support system ready for clinical implementation. Furthermore, although the incorporation of the *NBN* rs1805794 polymorphism yielded a statistically significant improvement in AUC (Δ = 0.076, 95% CI: 0.021–0.134; *p* = 0.008), this increase remains modest in absolute terms, and statistical significance should not be equated with clinical significance. Whether an improvement of this magnitude translates into a meaningful change in patient risk stratification or clinical decision making has not yet been established and would require prospective evaluation, ideally incorporating decision-analytic metrics such as net reclassification improvement or clinical utility analysis, before this genetic marker could be considered for routine clinical use.

Second, our genetic analysis was limited to a single polymorphism (*NBN* rs1805794). While incorporation of this variant improved model performance, other potentially relevant genetic factors were not evaluated. Future studies using genome-wide approaches may further refine genetic risk prediction for radiotherapy-related toxicity. However, incorporating genome-wide data into such predictive models poses considerable practical challenges. GWAS-based approaches typically require substantially larger sample sizes than candidate gene studies to achieve adequate statistical power, particularly given the need for stringent multiple testing correction across hundreds of thousands of variants. Furthermore, integrating high-dimensional genomic data with clinical and dosimetric variables within a single machine learning framework introduces additional complexity in terms of feature selection, model interpretability, and risk of overfitting, especially in cohorts of the size typically available in single-institution toxicity studies [45].

Beyond these methodological considerations, the practical implementation of genetic testing such as *NBN* rs1805794 genotyping into routine radiotherapy workflows would require careful evaluation of feasibility factors, including the cost of genotyping, accessibility of testing infrastructure across treatment centers, turnaround time relative to the radiotherapy planning timeline, and the logistics of integrating genetic results into existing clinical decision making pathways. Single-nucleotide polymorphism genotyping, such as AS-PCR used in the present study, is relatively low-cost and technically straightforward compared to genome-wide approaches; however, its incorporation into time-sensitive radiotherapy planning would still require dedicated laboratory infrastructure and standardized reporting pathways, which may not be readily available in all clinical settings. These practical considerations should be addressed in future implementation studies before genetic testing can be recommended as part of routine pretreatment risk assessment [46]. Another limitation of this study is that only disease-free survivors were included in the analysis. This inclusion criterion may have introduced a selection bias, as patients who experienced disease recurrence, progression, or early mortality—who may also be more likely to have developed severe treatment-related toxicities—were inherently excluded from the cohort. Consequently, the model’s findings may not be fully generalizable to the broader population of head and neck cancer patients treated with radiotherapy, particularly those with more aggressive disease courses or poorer overall prognosis. Finally as a further research direction, explainable AI (XAI) techniques such as SHapley Additive exPlanations (SHAP) could be incorporated into future iterations of this model. Compared with the partial dependence plots used in the present study, SHAP values provide patient-level, additive attributions of each feature’s contribution to an individual’s predicted risk, which may enhance clinician trust and facilitate the translation of model outputs into individualized clinical decision making. Addressing these limitations in future research will be crucial for further refining personalized radiotherapy protocols.

## 5. Conclusions

In conclusion, this exploratory study suggests that integrating clinical, dosimetric, and genetic information within a machine learning framework may offer a promising approach for predicting chronic treatment-related toxicity in patients with head and neck cancer. The proposed model demonstrated moderate predictive performance in this single-center cohort and should be regarded as a preliminary, hypothesis-generating step rather than a clinically applicable tool at this stage. Larger, external, and multi-institutional validation studies, along with broader genomic profiling, are needed before the clinical utility of such models can be established.

## Figures and Tables

**Table 1:** Clinical characteristics of the patients.

Parameter	Variable	n (%)
Gender	Female	33 (26.4)
	Male	92 (73.6)
Tumor location	Nasopharynx	81 (64.8)
	Larynx	16 (12.8)
	Hypopharynx	13 (10.4)
	Oropharynx	13 (10.4)
	Neck Metastasis of Unknown Primary	2 (1.6)
T stage	Tx	2 (1.6)
	T0	2 (1.6)
	T1	37 (29.6)
	T2	38 (30.4)
	T3	18 (14.4)
	T4	28 (22.4)
N stage	N0	34 (27.2)
	N1	21 (16.8)
	N2	50 (40)
	N3	20 (16)
Treatment schedule	RT	27 (21.6)
	CRT	55 (44)
	IC + CRT	43 (34.4)
Smoking history	Yes	69 (55.2)
	No	56 (44.8)
Continued smoking	Yes	31 (24.8)
	No	94 (75.2)
Diabetes mellitus	Yes	20 (16)
	No	105 (84)
RT technique	SIB	77 (61.6)
	Sequential	48 (38.4)

RT: radiotherapy; CRT: chemoradiotherapy; IC: induction chemotherapy; SIB: simultaneous integrated boost.

**Table 2:** Dosimetric parameters.

Parameter	Mean ± SD
PTV70 volume (cc)	118.6 ± 68.3
PTV60 volume (cc)	231.46 ± 148.5
PTV54 volume (cc)	641.1 ± 228.3
Oral cavity mean (Gy)	35.17 ± 13.7
Right submandibular mean (Gy)	52.4 ± 17.05
Left submandibular mean (Gy)	49.9 ± 17.1
Right parotid mean (Gy)	27.8 ± 11.3
Left parotid mean (Gy)	26.6 ± 10.3
Right temporomandibular joint mean (Gy)	38.7 ± 24.2
Left temporomandibular joint mean (Gy)	37.4 ± 23.7
Pharyngeal constructors mean (Gy)	53.49 ± 12.1
Pharyngeal constructors max (Gy)	69.6 ± 6.8
Esophagus max (Gy)	52.8 ± 15.4
Larynx mean (Gy)	46.05 ± 15.5
Thyroid mean (Gy)	47.3 ± 14.3
Left cochlea mean (Gy)	29.2 ± 20.3
Right cochlea mean (Gy)	29.9 ± 20.5

**Table 3:** Chronic side effects in the patient cohort.

Parameter	Variable	n (%)
Dysgeusia	No	97 (77.6)
	Yes	28 (22.4)
Tooth loss	No	39 (31.2)
	Yes	86 (68.8)
Chronic otitis	No	52 (41.6)
	Yes	73 (58.4)
Trismus	No	94 (75.2)
	Yes	31 (24.8)
Xerostomia	No	31 (24.8)
	Grade 1	62 (49.6)
	Grade 2–3	32 (25.6)
Neck fibrosis	No	55 (44)
	Grade 1	34 (27.2)
	Grade 2–3	36 (28.8)
Dysphagia	No	81 (64.8)
	Grade 1	31 (24.8)
	Grade 2–3	13 (10.4)
Aspiration	No	84 (67.2)
	Grade 1	24 (19.2)
	Grade 2–3	17 (13.6)
Hypothyroidism *	No	76 (65.0)
	Yes	41 (35.0)
Ocular side effects	No	115 (92)
	Yes	10 (8)
Hearing loss	No	35 (28)
	Grade 1	33 (26.4)
	Grade 2	26 (20.8)
	Grade 3	31 (24.8)
Hypopituitarism	No	121 (96.8)
	Yes	4 (3.2)
Carotid artery stenosis	No	105 (84)
	Yes	20 (16)
Osteoradionecrosis	No	124 (99.2)
	Yes	1 (0.8)

* Eight patients with pre-existing hypothyroidism prior to radiotherapy were excluded from the hypothyroidism analysis; percentages for this variable are calculated based on the remaining 117 patients.

**Table 4:** *NBN* rs1805794 genotypes and allele frequencies.

Variant	Genotype	Genotype Distribution n (%)	Allele	Allele Frequency	pHWE
rs1805794	GG	51 (40.8)	G	0.632	0.680
	GC	56 (44.8)	C	0.368	
	CC	18 (14.4)			

**Table 5:** Performance comparison of machine learning classifiers without genomic data.

Classifier	AUC	ACC	Sensitivity	Specificity	Precision	F1 Score	MCC
Logistic Regression	0.665 ± 0.020	0.645 ± 0.018	0.620 ± 0.019	0.670 ± 0.017	0.640 ± 0.018	0.630 ± 0.017	0.620 ± 0.019
SVM	0.675 ± 0.021	0.655 ± 0.019	0.630 ± 0.020	0.680 ± 0.019	0.650 ± 0.020	0.640 ± 0.018	0.630 ± 0.020
Random Forest	0.695 ± 0.019	0.675 ± 0.017	0.650 ± 0.017	0.700 ± 0.017	0.670 ± 0.018	0.660 ± 0.017	0.650 ± 0.019
XGBoost	0.705 ± 0.020	0.685 ± 0.018	0.660 ± 0.019	0.720 ± 0.018	0.680 ± 0.019	0.670 ± 0.018	0.660 ± 0.020

AUC: Area under curve, ACC: Accuracy, MCC: Matthews correlation coefficient.

**Table 6:** Performance comparison of machine learning classifiers with genomic data (*NBN* rs1805794 polymorphism).

Classifier	AUC	ACC	Sensitivity	Specificity	Precision	F1 Score	MCC
Logistic Regression	0.712 ± 0.015	0.708 ± 0.014	0.680 ± 0.014	0.730 ± 0.015	0.700 ± 0.015	0.690 ± 0.014	0.680 ± 0.015
SVM	0.732 ± 0.016	0.728 ± 0.015	0.700 ± 0.016	0.750 ± 0.017	0.720 ± 0.018	0.710 ± 0.016	0.700 ± 0.017
Random Forest	0.758 ± 0.012	0.750 ± 0.013	0.720 ± 0.011	0.770 ± 0.012	0.740 ± 0.013	0.730 ± 0.012	0.720 ± 0.014
XGBoost	0.781 ± 0.011	0.770 ± 0.014	0.740 ± 0.015	0.790 ± 0.016	0.760 ± 0.017	0.750 ± 0.015	0.740 ± 0.013

AUC: Area under curve, ACC: Accuracy, MCC: Matthews correlation coefficient.

## Data Availability

The data that supports the findings of this study are available from the corresponding author upon reasonable request.
